# A phylogenetic analysis of macroevolutionary patterns in fermentative yeasts

**DOI:** 10.1002/ece3.2097

**Published:** 2016-05-10

**Authors:** Rocío Paleo‐López, Julian F. Quintero‐Galvis, Jaiber J. Solano‐Iguaran, Angela M. Sanchez‐Salazar, Juan D. Gaitan‐Espitia, Roberto F. Nespolo

**Affiliations:** ^1^ Instituto de Ciencias Ambientales y EvolutivasUniversidad Austral de ChileValdivia5090000Chile; ^2^ CSIRO Oceans & AtmosphereGPO Box 1538Hobart7001TasmaniaAustralia; ^3^ Center of Applied Ecology and Sustainability (CAPES)Facultad de Ciencias BiológicasUniversidad Católica de ChileSantiago6513677Chile

**Keywords:** Adaptive radiation, comparative method, fermentation, phylogenetic signal, Saccharomicotina

## Abstract

When novel sources of ecological opportunity are available, physiological innovations can trigger adaptive radiations. This could be the case of yeasts (Saccharomycotina), in which an evolutionary novelty is represented by the capacity to exploit simple sugars from fruits (fermentation). During adaptive radiations, diversification and morphological evolution are predicted to slow‐down after early bursts of diversification. Here, we performed the first comparative phylogenetic analysis in yeasts, testing the “early burst” prediction on species diversification and also on traits of putative ecological relevance (cell‐size and fermentation versatility). We found that speciation rates are constant during the time‐range we considered (ca., 150 millions of years). Phylogenetic signal of both traits was significant (but lower for cell‐size), suggesting that lineages resemble each other in trait‐values. Disparity analysis suggested accelerated evolution (diversification in trait values above Brownian Motion expectations) in cell‐size. We also found a significant phylogenetic regression between cell‐size and fermentation versatility (*R* ^2^ = 0.10), which suggests correlated evolution between both traits. Overall, our results do not support the early burst prediction both in species and traits, but suggest a number of interesting evolutionary patterns, that warrant further exploration. For instance, we show that the Whole Genomic Duplication that affected a whole clade of yeasts, does not seems to have a statistically detectable phenotypic effect at our level of analysis. In this regard, further studies of fermentation under common‐garden conditions combined with comparative analyses are warranted.

## Introduction

Evolutionary innovations can trigger diversification (adaptive radiations: sudden increases in speciation rates (Yoder et al. 2010), ecological success of a single lineage or a combination of both (the ecological success is followed by later speciation and diversification). As a consequence of the later phenomenon, it is usually possible to identify entire clades bearing an innovation (Pincheira‐Donoso et al. 2015). Among microorganisms, a probable case of adaptive evolution is yeasts and fermentation, where a physiological innovation related to the capacity to extract energy from single sugars, seems to have triggered a major adaptive change (see reviews in Dashko et al. 2014; Hagman and Piskur 2015). Whether this phenomenon fall in one of the situations described before, is unclear.

Phylogenies can give information about speciation patterns (the topology) and time of divergence among species (branch lengths), that can be assessed with simple speciation models, such as the rate of species production in time (Nee 2006). When this information is put together with trait values, several patterns and processes that are not evident from qualitative assessments can emerge. For instance, the distribution of character states can be compared across basal and derived nodes, which permits the inference of losses and acquisitions (Revell 2012). On the other hand, simply asking whether lineages resemble each others more or less than what is expected by a Brownian Motion model (BM) of evolution, reveals whether homogenizing or diversifying processes were important (Blomberg et al. 2003). Trait diversification, analyzed in the time elapsed from the most basal node in the phylogeny to the actual species (i.e., the “tips”), and compared with BM expectations indicate whether a trait showed peaks of diversification, concomitant with species diversification (Harmon et al. 2003). In this context, correlated evolution between traits can be analyzed using the phylogeny as a part of the process (i.e., phylogenetic regression models; Pollux et al. 2014).

Yeasts (Ascomycota:Saccharomycotina) are a monophyletic lineage of unicellular fungi that counts over thousand species (James et al. 2006; Kurtzman et al. 2011). Wild yeasts live as saprobes, often in the interface between plants and animals, but an important number are domesticated for industrial processes such as baking, brewing and synthesis of recombinant proteins. Although best known by their capacity to produce and metabolize ethanol, the diversity of substrates metabolized by yeasts is enormous. Yeasts can metabolize sugars (BM e.g., sucrose, galactose, trehalose, maltose), heavy metals (e.g., Cu, Zn, Cd), aromatic compounds (e.g., catechol, vanillin) and nitrates (Kurtzman et al. 2011). This makes them of commercial, ecological and medical relevance, and at the same time have generated a rich nomenclature of standardized responses to biochemical and physiological tests, including fermentation (Kurtzman et al. 2011). Here, we were focused on the maximum number of sugars a species can ferment, a trait that we defined operationally as “fermentation versatility”. We are aware that this trait definition is blind to the specific fermented sugar, which could limit our conclusions. However, this is a good proxy of the metabolic machinery that permits (or constraint) the maximum fermentative capacity.

**Figure 1:**
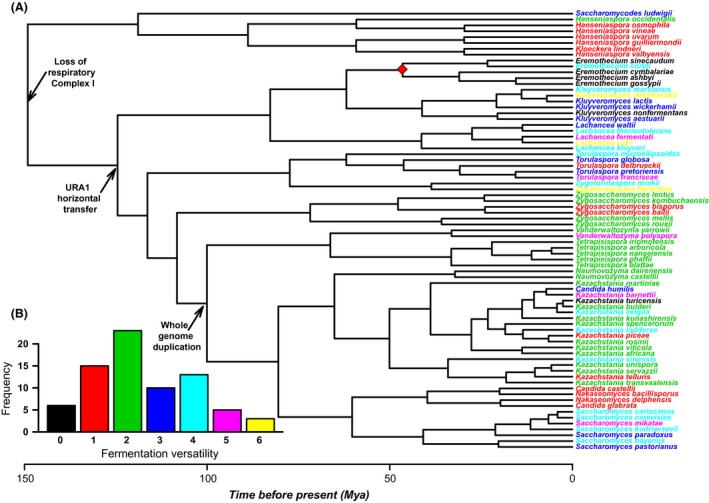
(A) Our working phylogeny, modified from Kurtzman and Robnett, 2003 with actualized species names according to Kurtzman et al. 2011). Fermentative versatility (maximum number of sugars a species can ferment) was mapped as different text colors (0 = no fermentation; 6 = the species can ferment six different sugars). Three major evolutionary events are depicted, which were used for calibrating the phylogeny: the whole genomic duplication (100 MYA), the horizontal gene‐transfer of the URA1 gene from bacteria (125 MYA) and the loss of respiratory complex I from mtDNA (150 MYA; see (Dujon 2010). (A) The red diamond indicates, according to Hagman et al. (2013), absence of the URA1 gen in this clade (this is debated). (B) Distribution of fermentative capacity in the phylogeny (most species can ferment two sugars).

**Figure 2:**
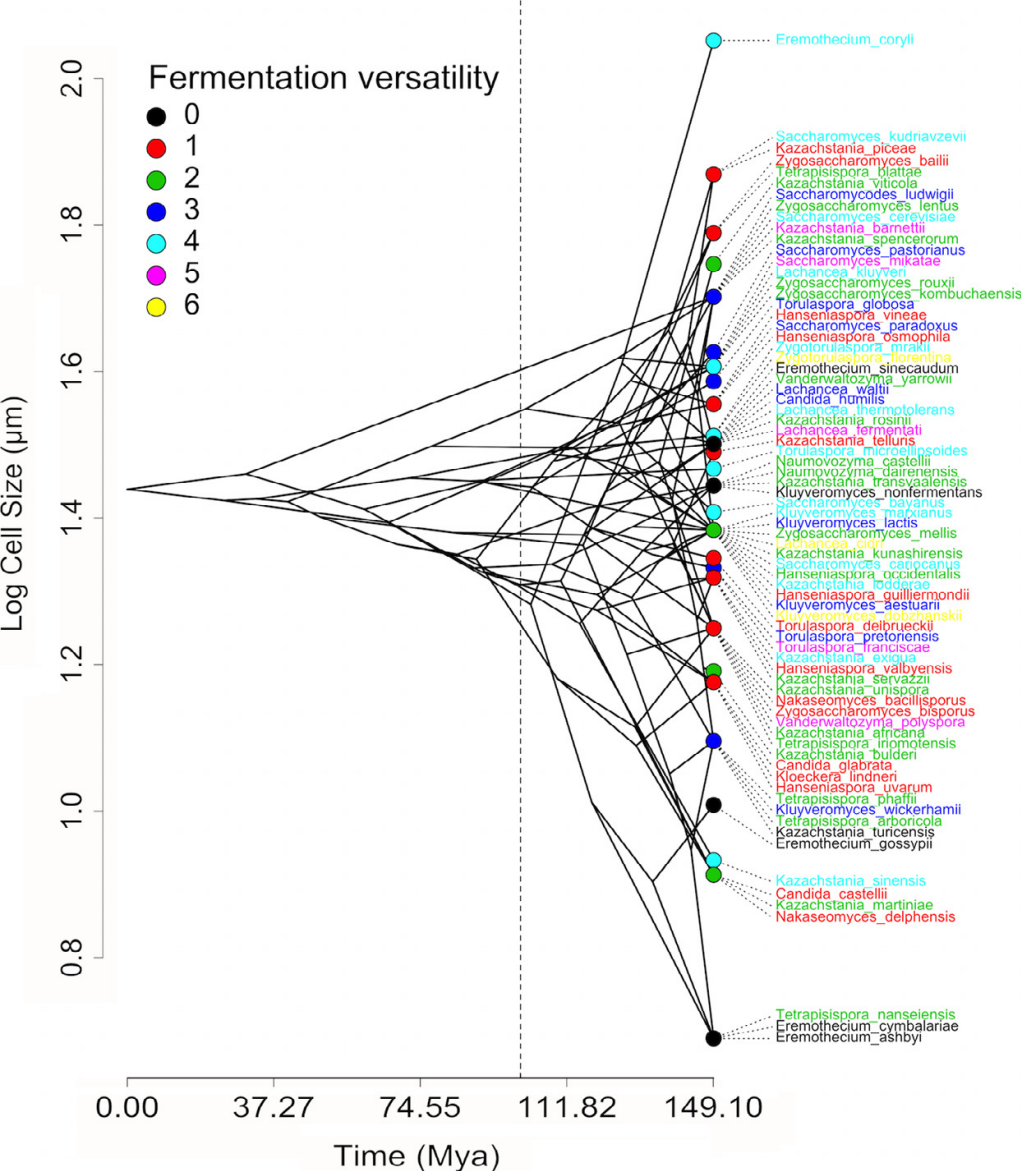
Phenogram showing the diversification of cell‐size across time, in our working phylogeny. The dotted line indicates, approximately, the Whole Genome Duplication that occurred 100 MYA (see the text for details).

The distribution of fermentation versatility (trait values from Kurtzman et al. 2011; see Material and Methods) on a yeast's phylogeny is depicted in Figure 1A, together with some important genomic rearrangements that yeasts experienced during the last 150 millions of years (reviewed in Dujon 2010; Hagman et al. 2013; Dashko et al. 2014). These events are important because it is hypothesized that they were relevant in determining their fermentative capacity (particularly the Whole Genomic Duplication [WGD]), by increasing the relative dosage of glycolytic genes, thereby increasing flux through the glycolysis pathway and providing polyploid yeasts with a growth advantage through rapid glucose fermentation (Conant and Wolfe 2007). Three facts are evident from this figure (Figure 1): first, some entire clades show similar phenotypic characteristics (e.g., *Tetrapisispora*, which ferment two sugars) but some others show high intraclade variation (e.g., *Saccharomyces,* fermenting from three to six sugars, see Fig. 1A). Second, it is almost impossible, without a statistical test, to determine in what degree lineages resemble each other in trait values. Third, historical (genomic rearrangements, in this case) events cannot be unambiguously associated to a given phenotypic value (although many authors claim the contrary, reviewed by Dashko et al. 2014). A similar visualization in a continuous trait such as cell size (a “phenogram”) is presented in Figure 2. Here, it can be seen that the species with largest cell‐size is *Eremothecium coryli*, the sole fermentative species of this genus (it ferments three sugars, the remainding four do not ferment any sugar, see Fig. 1A, species in black text). This qualitative pattern suggest that comparatively larger cells can ferment more sugars, but large intraclade variation in both characters preclude any firm conclusion (for instance, the lowest in cell‐size ranking is *Tetrapisispora nanseiensis*, which ferment two sugars, see Fig. 1A). Therefore, some metrics are needed to treat this variation at the phylogenetic level.

Phylogenetic signal (PS), which is at the core of modern phylogenetic analysis, is a measure of how much species resemble each other in trait values (Blomberg et al. 2003; Munkemuller et al. 2012; Paradis 2011). In other words, it gives an idea, on average, of how a given trait followed the topology of a phylogeny in its diversification, assuming that this diversification followed a BM model of evolution (Pennell et al. 2014). One of the most used metrics for PS is the *K*‐statistic (Blomberg et al. 2003), see examples in (Munkemuller et al. 2012; Fisher et al. 2013; Gingras et al. 2013; Wang and Clarke 2014) which is computed from the phylogenetic variance‐covariance matrix (vcv; see Material and Methods), when *K* = 1 the trait variation is equal to BM expectations (Blomberg et al. 2003). If a phylogeny was built based on morphological data, then the PS of a morphological trait in this phylogeny will be 1.0.

According to a number of authors, the appearance of fruits coincided with the appareance of fermentative yeasts (especially *Saccharomyces*), which has supported the hypothesis of novel ecological niches provided by fruits and simple sugars (Hagman et al. 2013; Dashko et al. 2014; Williams et al. 2015). These authors suggested that fermentative capacity represents an ecological innovation that triggered an adaptive radiation. One prediction of adaptive radiation theory is to observe an inflection in speciation rates, at some point in time (“early bursts”, see examples in Rabosky et al. 2014). The theory also predicts that ecologically relevant traits should show early increases in diversification, followed by decelerated evolution (related to BM expectations). We chose cell‐size, as an ecologically relevant trait (Jiang et al. 2005; Yoshiyama and Klausmeier 2008; Turner et al. 2012), which in eukaryotic unicellular organisms is known to determine the capacity to process different compounds (the larger the cell, the more intracellular compartments it can have, see Raven et al. 2005; Nakov et al. 2014).

We are not aware of a single study applying comparative phylogenetic methods to study the evolution of yeasts. Consequently, we present this preliminary comparative analysis, involving a phylogeny, traits and time calibration with the aims of: (1) testing the prediction of adaptive radiation theory in diversification rates (“early bursts”) in Saccharomycotina, (2) testing if fermentation versatililty and cell‐size experienced correlated evolution, (3) to explore whether WGD and non‐WGD species show detectable phenotypic differences at our level of analysis and (4) to determine whether a model assuming an evolutionary optimum in cell‐size is more probable than alternative models. If WGD had consequences at the phenotypic level, we should be capable of detecting them as different evolutionary optima.

## Material and Methods

### Data compilation and phylogeny

We re‐compiled the phylogeny of Kurtzman and Robnett (2003) with guidelines provided by the first author. This phylogeny was obtained using four nuclear genes (large subunit rRNA, small subunit, ITS‐5.8S and translation elongation factor‐1*α*) and two mitochondrial genes (mitochondrial SS rRNA and COXII; Kurtzman and Robnett 2003). The methods for phylogenetic reconstruction were reported earlier (Kurtzman and Robnett 2003; see updates in Kurtzman et al. 2011). Briefly, we downloaded the sequences reported by the author to obtain the phylogenetic relatedness among species, using the maximum likelihood (ML) function included in MEGA v6 (Tamura et al. 2013). Bootstrap support for ML was determined from 1000 replicates. The phylogeny was trimmed according to the availability of phenotypic data. For each species, we compiled cell size and fermentation versatility (Kurtzman et al. 2011).

The statistical analyses were performed with the original phylogeny, which included branch lengths in genetic distances. As long as these distances are linearly constant across lineages (which is the case with genetic data assuming a molecular clock), results will be similar as done on a time‐calibrated phylogeny (Paradis 2011). Still, we time‐calibrated the phylogeny using three different historical events: the loss of the respiratory complex I, which occurred 150 millions of years ago (MYA; Marcet‐Houben et al. 2009), the horizontal transfer of the URA1 gene, which according to Dujon (2010) occurred 125 MYA and the WGD, which according to Wolfe et al. (1997) occurred 100 MYA (Fig. 1A). The calibration was performed with the chronopl command in ape (Paradis 2011).

For cell‐size, we considered the average cell‐size of the lower diameter reported for each species, because most yeast cells are asymetric (see the phenotypic distribution in Fig. 2; Rupes 2002). For fermentation versatility, we considered the seven most common sugars metabolized by all species (glucose, galactose, sucrose, maltose, lactose, raffinose, and trehalose). We considered all positive (+) fermentation tests reported for each species, together with those codified as “s” (slow response) and “w” (weak response). Species codified as with a “variable” response (“±” or “v”) were considered as negative (see Kurtzman et al. 2011, pp. 223–277). This variable was transformed to an ordinal scale that goes from zero (no‐fermentative capacity) to six (the species can ferment six different sugars; see character mapping in Fig. 1A).

The complete dataset and the phylogeny (as text file) is provided as Supplementary information, together with a R‐script that runs all the analyses in the order presented in Results.

### Phylogenetic methods

We performed a combination of phylogenetic analyses: descriptive phenotypic mapping; PS; diversification analysis in species and traits (disparity); analysis of cell size diversification throughout the phylogeny of yeast; and the analysis of correlated evolution using phylogenetic regressions (to explore correlated evolution between cell‐size and fermentation versatility).

#### PS (transition rates and Blomberg's *K*)

For analyzing PS (i.e., how lineages resemble each other in trait values) in the discrete trait (fermentation versatility), we used the phylo.signal.disc procedure (E. Rezende, pers.comm., available script on request). This algorithm estimates the minimum number of character‐state transitions at each node that account for the observed distribution the character in the phylogeny (assuming maximum parsimony; Maddison and Maddison 2000). Then, it is compared with the median of a randomized distribution (1000 randomizations were used). If the observed transition rates are significantly less than the randomized median, a significant PS is inferred. The PS of the continuous trait (lograrithm of cell‐size) was estimated using the *K*‐statistic (Blomberg et al. 2003; Munkemuller et al. 2012). The *K*‐statistic is an adimensional index, obtained from the phylogenetic vcv, which summarizes the distance information of a given phylogeny. The diagonal contains all root‐to‐tip distances for each species, and in the off‐diagonal contain all distances from the most recent common ancestor (see details of the vcv matrix construction in Swenson 2014, p. 157; and also in Blomberg et al. 2003). Then, *K* is computed as *K* = [observed (MSEo/MSE)]/[expected (MSEo/MSE)], where MSEo are the observed mean‐squared errors and MSE are the expected mean‐squares under a BM model of evolution. In this way, *K* = 1.0 represents trait evolution as expected by a BM model for evolution (BM), and values below unity mean that lineages resemble each other less than what expected by BM. The significance of *K* (null hypothesis being *K* = 0) was tested by comparing (in a ratio) the observed variance of trait's independent contrasts, to the variance of independent contrasts obtained by a randomization (see details in Blomberg et al. 2003; Swenson 2014).

#### Diversification analysis

We performed preliminary explorations of speciation rates using Bayesian Analysis of Macroevolutionary Mixtures (BAMM, versión 2.2.0; Rabosky et al. 2014). In brief, this procedure uses reversible jump Markov chain Monte Carlo to automatically explore a vast universe of candidate models of lineage diversification and trait evolution, and then mapping the most likely speciation rates in the phylogeny (“phylorate” plots) and over time (specitation‐through‐time plots). Rate‐shifts, the consequences of adaptive radiations, are readily observed in these plots as a slow‐down in speciation rates.

#### Disparity analysis and cell size diversification dynamics

To determine how log (cell size) evolved across the phylogeny, we plotted disparity‐through‐time (dtt) using the dtt function in the geiger in R (Harmon et al. 2003; Swenson 2014). Disparity was calculated from average pairwise Euclidean distances between species, a variance‐related method of estimating the dispersion of points in multivariate space that is insensitive to sample size. Disparity is calculated for the entire clade and then for each subclade defined by a node in the phylogeny. Relative disparities for each subclade were standardized by dividing a subclade's disparity by the disparity of the entire clade. The patterns of disparity through time were calculated by moving the phylogeny from the root. At each divergence event (i.e., each node), we calculated the mean relative disparity for that point in time as the average of the relative disparities of all subclades whose ancestral lineages were present at that time (Harmon et al. 2003). The disparity of daughter nodes are usually compared with a null distribution generated by simulating trait evolution on the phylogeny, many times under BM (see details in Harmon et al. 2003; Swenson 2014, p. 170). A metric of the rate of trait evolution from dtt plots, is the morphological disparity index (MDI). The MDI is calculated as the area between the observed disparities connected by a line, and the median of the expected disparities obtained from the BM simulations, in a dtt plot (Swenson 2014). Negative values of MDIs are interpreted as early burst of in the evolution of trait diversity, followed by little diversification within more terminal subclades. Conversely, positive MDIs are taken as evidence of a constant or accelerating rate of trait diversification. To further discriminate between “early” versus “late” bursts hypotheses, we fitted four alternative models using the geiger package. Namely, the Brownian‐motion model (BM) describing trait evolution based on random walk processes, which assumes that trait variance is centered around the initial value at the root of the tree, and increases proportionally to the distance from the root. Second, the Ornstein‐Uhlenbeck model (OU), which assumes that once traits have evolved through stabilizing selection, they are pulled to an adaptive optimum (Butler and King 2004). Third, the Early‐Burst model (EB), which describes exponentially increasing or decreasing rates of evolution over time, assuming the greatest phenotypic divergence (Harmon et al. 2010). With the EB model, the “*a*” parameter indicate whether divergence was early (large values) or late (small values) (Harmon et al. 2010). The EB model is also known as the ACDC (accelerating‐decelerating model of Blomberg et al. 2003), and fits a model where the rate of evolution increases or decreases exponentially through time: *r*[*t*] = *r*[0] × exp(*a* × *t*), where *r*[0] is the initial rate, *a* is the rate change parameter, and *t* is time. The maximum bound was set to −0.000001, representing a decelerating rate of evolution. For accelerate rates of evolution, we set this bound to 5. Finally, we fit a white‐noise (non‐phylogenetic) model, assuming data coming from a single normal distribution with no covariance structure among species (Harmon et al. 2008). Then, to determine whether WGD and non‐WGD species show different evolutionary optima in log (cell‐size), we used the OWie package to adjust a BM model (BM1), a model assuming a single optimum (OU1), a BM model assuming a multiple rates (BMS) and a model assuming multiple optima (OUM).

Comparisons of goodness of fit and selection of the best evolutionary models were performed through the Akaike information criterion for small simple size (AICc) (Burnham and Anderson 2002; Dlugosz et al. 2013). All these analyses were performed using the geiger package.

#### Phylogenetic regression

To test if (log transformed, because this improved distribution properties) of cell‐size was correlated with fermentation versatility, we applied ordinary least‐squares and generalized linear models using the variance‐covariance structure of the phylogeny (with the internal function corPagel; GLS). We used the AICc model selection criterion, as explained before, to choose the best model.

## Results

The distribution of trait values suggests that most species ferment two sugars, being zero and six, extreme character states (Fig. 1B). This representation shows the occurrence of multiple loses and acquisitions in fermentative versatility (Fig. 1A). For instance, the capacity to ferment six sugars seems to have appeared independently in *Lachancea cidri* and *Zygotorulaspora florentina* (yellow in Fig. 1A). On the other hand, fermentative capacity seems to have been lost in *Kluyveromyces nonfermentans* and *Kazachstania turicensis* (Fig. 1A). Similarly, the phenogram of cell‐size diversification suggests this trait diversified rather recently, with some coincidence with the WGD that affected yeasts about 100 MYA (Fig. 2).

**Figure 3:**
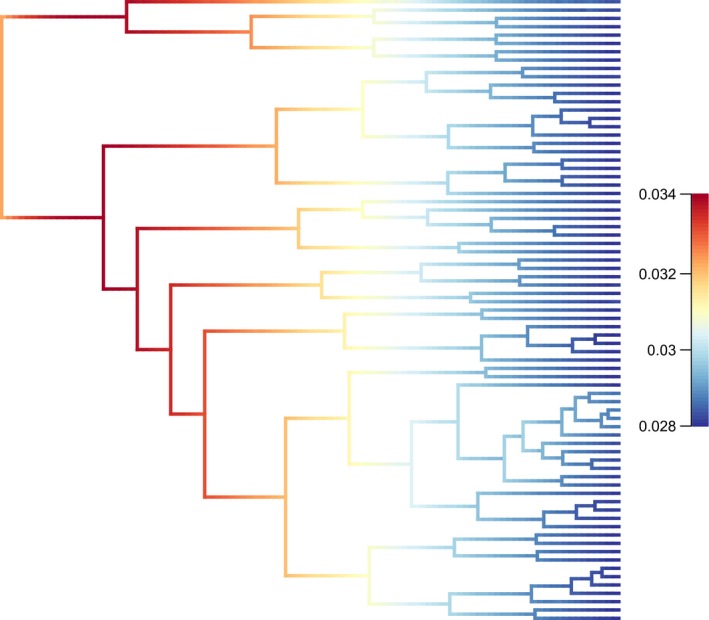
A phylorate plot: speciation dynamics during the evolution of yeasts. This plot shows the most probable shift configuration sampled with BAMM. Warmer colors denote faster rates of speciation. Rate values represent new lineages per million years. The posterior probability of this tree is 98%. A radiation would have been observed as a terminal clade in red, which is not the case.

**Figure 4:**
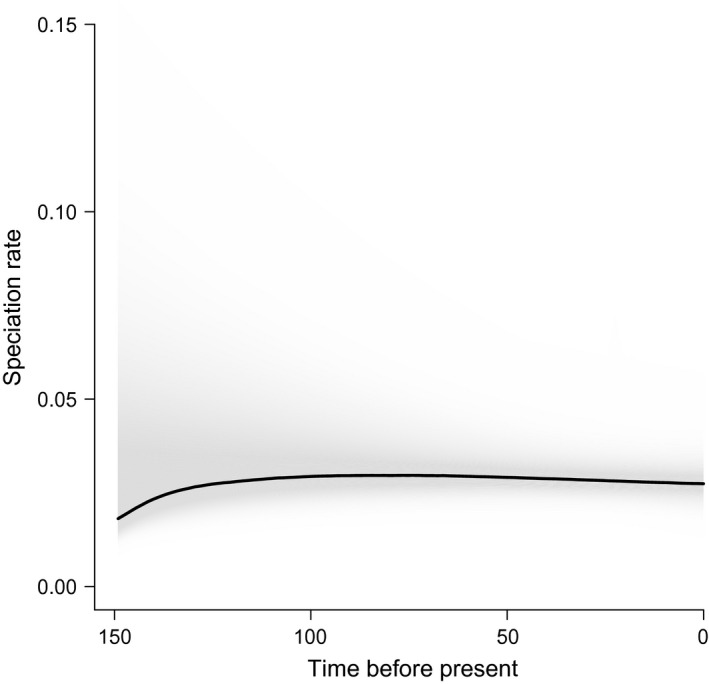
Speciation‐through‐time trajectory for the yeast phylogeny, as calculated from BAMM (see Fig. 3).

Fermentation versatility showed significant PS: there were 35 observed transitions, and the randomized median was 45 (*P* < 0.001). For log (cell‐size), the *K*‐statistic was significant but lower than expected by BM (*K* = 0.25; *P* = 0.009; BM expectations: *K* = 1.0). Speciation rates, as shown by the bamm plot (Fig. 3; blue: low speciation rates; red: high speciation rates) do not change much across the phylogeny; there is no evidence of rate‐shifts on any branch. Evolutionary rates through time also show no rate‐shifts in speciation rates and there is no signature of decreasing rates through time, thus providing no support for the early burst hypothesis (Fig. 4).

**Figure 5:**
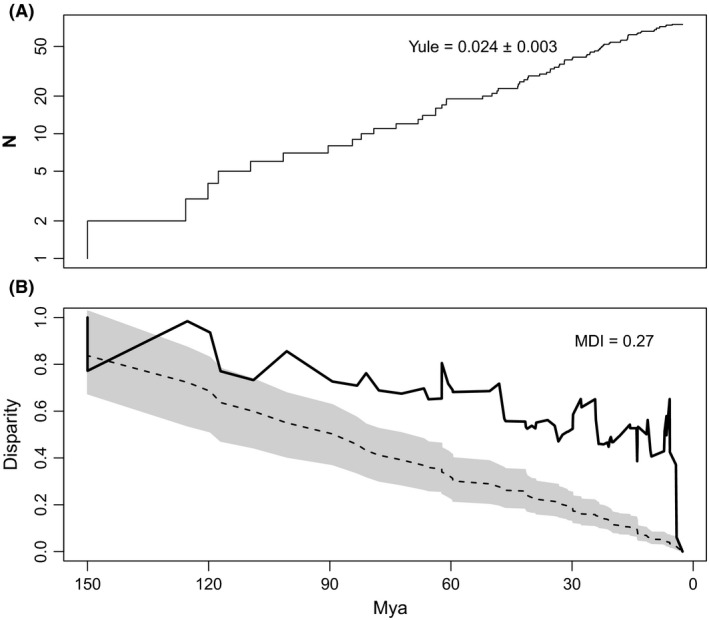
(A) Lineage‐through‐time plot showing the production of species from the last common ancestor. The time‐scale obtained from the calibration is explained in Figure 4. Speciation rate, assuming a pure‐birth model (“Yule”) is shown. (B) Disparity‐through‐time plot showing observed disparity (a measure of phylogenetic signal at each node of the phylogeny) in the solid line, compared with the expected value under Brownian Motion (dotted line) and a null distribution of 999 randomizations (shaded area showing 95% interval). The morphological disparity index (MDI = 0.27) was significantly different after comparing it with a null distribution (*P* = 0.001).

The ltt‐plot shows the typical pattern of lineage increase in time, with a speciation rate of 0.024 (Fig. 5A). The dtt‐plot reveals that cell‐size disparity is partitioned within subclades far more than expected under BM (MDI = 0.27, *P* = 0.001; Fig. 5B). This result is consistent with the scenario of accelerated evolution in this trait and contrary with the idea of early bursts (see Discussion).

The model‐based analysis using AICc (AIC corrected for small sample‐size) for (log) cell size diversification suggested the best model, compared with alternative evolutionary models including white‐noise is OU (single optimum; Table 1). More specifically, the OWie analysis for testing whether one or multiple optima are best descriptors of the data showed that the best model is OU1 (Orstein‐Uhlenbeck with single optimum, Table 2), which do not support the hypothesis that WGD and non‐WGD species differ in log (cell‐size). Also, this evidence does not support the prediction of early bursts in cell‐size diversification, which is confirmed by the dtt‐plot (Fig. 5B), the positive MDI and by the phenogram suggesting late diversification in this trait (Fig. 2).

**Table 1:** The output of the fitContinuous command

	AICc	AICw
BM	33.923	0
OU	12.968	0.973
EB	36.095	0
DL	22.034	0.010
WN	21.131	0.016

AICc values (smaller is better) according to different models of evolutionary diversification in log (cell‐size). The best model (i.e., near unity) is the one with the highest Akaike weight, and is underlined. BM, Brownian Motion; OU, Orstein‐Uhlenbeck with a single optimum; EB, early burst; DL, delta model; WN, white noise.

**Figure 6:**
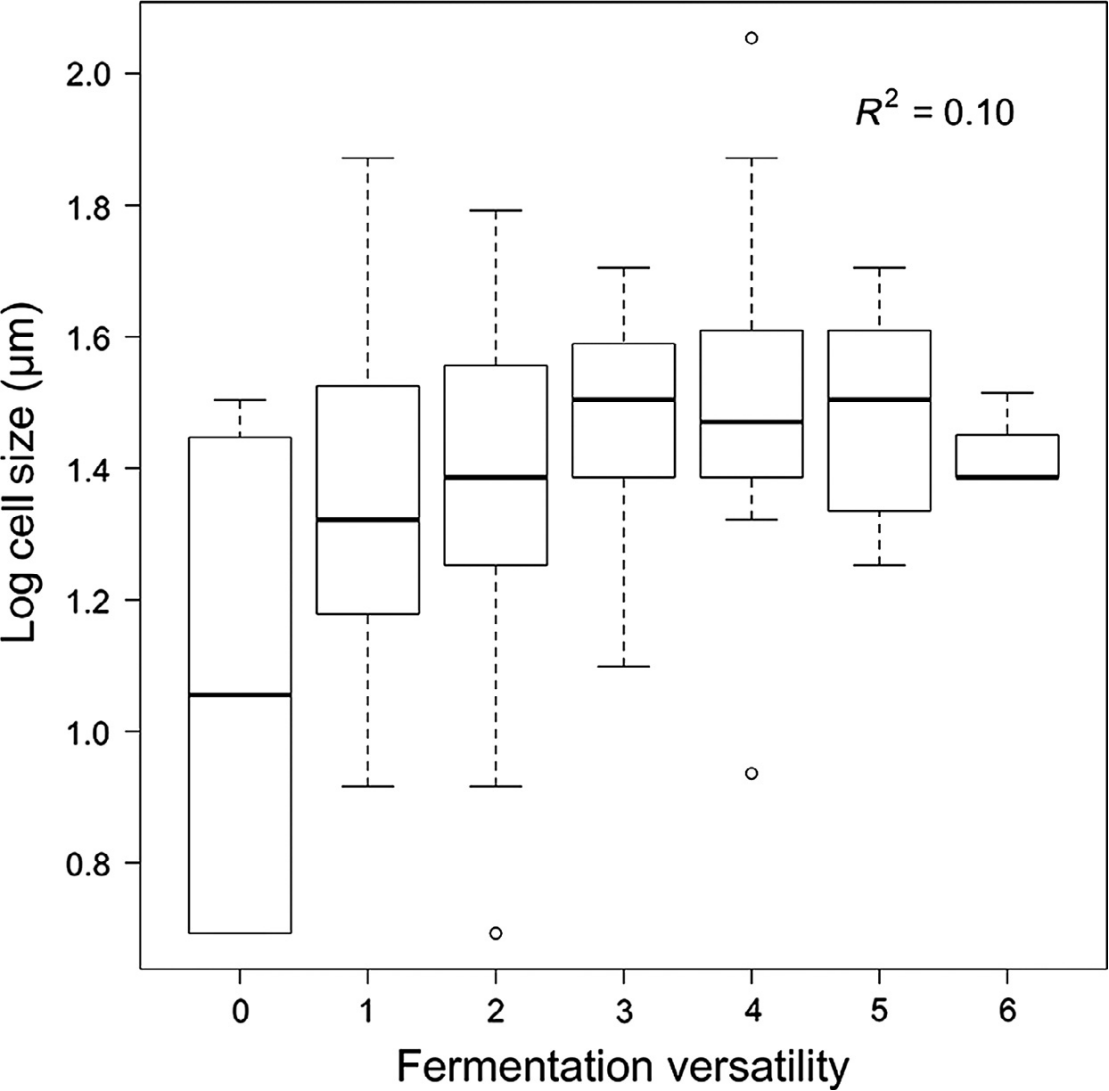
Relationship between double‐log of cell size and fermentation. The relationship was significant after a GLS analysis including phylogenetic relationships. The model considering the phylogeny ranked significantly better than a model assuming no phylogenetic relationships (a “star” phylogeny, see Results for details and statistics).

The AICc score for the model of phylogenetic regression between log‐log (cell‐size) and fermentation was ranked better (AICc = −35.1, df = 4) than a model without phylogenetic structure (a “star” phylogeny; AICc = −20.7, df = 3). The model including phylogenetic structure showed significant effects of fermentation versatility, on log [cell‐size]) (*R* ^2^ = 0.10, *P* = 0.017). This suggests a significant correlation between cell size and fermentation versatility, after accounting for phylogenetic relatedness (Fig. 6).

**Table 2:** The output of the OWie command

	lnL	AICc	dAICc	AICwi
BM1	−14.878	33.923	20.955	0
OU1	−3.315	12.968	0	0.659
BMS	−10.271	26.881	13.913	0.001
OUM	−2.858	14.288	1.320	0.341

AICc values (smaller is better) according to different models of evolutionary diversification in log (cell‐size). The best model (i.e., near unity) is the one with the highest Akaike weight, and is underlined. BM1, Brownian Motion; OU1, Orstein‐Uhlenbeck with a single optimum; BMS, Brownian Motion with multiple rates; OUM, Orstein‐Uhlenbeck with multiple optima, according to WGD and non‐WGD species (see text for details).

## Discussion

Examples of physiological changes that had profound effects on the evolutionary history of a lineage are well‐described in vertebrates (Berenbrink et al. 2005; Shen et al. 2010), invertebrates (Bond and Opell 1998), and plants (Crayn et al. 2004; Jobson et al. 2004). In all these cases, a key innovation associated with a novel ecological niche, performance or function was identified: higher metabolic capacity for flight in bats, oxygen secretion capacity for buoyancy in teleosts, or viscous adhesive threads in spiders (Bond and Opell 1998; Berenbrink et al. 2005; Shen et al. 2010). In this study, we explored whether yeast evolution is such a case, by testing a basic prediction of adaptive radiation theory: the existence of early bursts in trait and species diversification (Pincheira‐Donoso et al. 2015). To attain this, we applied comparative phylogenetic methods, particularly those related with the graphic characterization of speciation rates in time (bamm plots and lineage‐through‐time plots), model selection and disparity analysis applied to cell‐size. With the chosen traits and phylogeny, we did not find evidences of such early bursts, not supporting the existence of an adaptive radiation in the lineage of yeasts. Such evidence would have been evident in the bamm plot (Fig. 2) as a slow‐down in speciation rate, as a early inflection in the speciation curve (Fig. 3) (see examples in Rabosky et al. 2014), as a negative MDI in the disparity analysis of cell‐size (see example in Colombo et al. 2015), or after the AICc analysis for different evolutionary models.

It could be argued that given the large population sizes and short generation times of yeasts, comparing traits among species across the geological time‐scale would not be sufficiently sensitive to infer adaptive patterns (Goddard and Grieg 2015). Based on this study, as well as others (e.g., adaptation to pH in archaea: Gubry‐Rangin et al. 2015; adaptations to cactus environments in yeasts: Starmer et al. 2003; adaptations to salt water in diatoms: Nakov et al. 2014), we believe the contrary. As long as a lineage (described by a specific collection of molecular, reproductive and/or phenotypic criteria) can survive and proliferate in an environment defined by a number of precisely demarcated conditions, adaptation to an ecological niche could be detected. Excedingly high speciation rates can be a problem, but this is something that can be calculated from the phylogeny. For instance, our estimations of speciation rates in yeasts were around 0.02 (Fig. 5A), which are small, compared with other groups (Table 3).

**Table 3:** Speciation rates (pure birth model) calculated from different available phylogenies

Taxa	Speciation rate (±SE)	*N*	Reference
Birds	0.058 ± 0002	915	Jetz et al. (2012) (subset)
Mammals^a^	7.98 ± 0.62	169	Meredith et al. (2011)
Diatoms	0.026 ± 0.0017	247	Sorhannus (2007)
Bacteria^a^	64.1 ± 2.21	841	Lang et al. (2013)
Yeasts^a^	0.024 ± 0.003	77	Kurtzman and Robnett (2003) (used in this study)

Numbers are scaled to the length of the whole phylogeny (i.e., they are comparable).

a The phylogenetic tree was made ultrametric using the command chronopl(tree, 1) (Paradis 2011).

The significant PS of fermentation versatility suggests that this variable is explained by phylogenetic relationships (several whole clades share character states). However, the qualitative assessment of trait mapping also indicates that losses and acquisitions are common, suggesting that increasing or reducing the number of sugars that a species can ferment is relatively easy. How this occurs is unclear, but points to changes in sugar transporter as a fundamental process.

The incorporation and use of a broad repertoire of sugars during yeast evolution is probably the result of mutations on some of the sugar transporter genes and the existence of a multigene family of sugar carriers (Bisson et al. 1993). The complex interactions of these genes can regulate glucose repression (Carlson 1999) and allow the acquisition and metabolization of different fermentable carbon sources (Weinhandl et al. 2014). For instance, by mean of the GAL network, yeast cells can use galactose or other available carbon sources (Stockwell et al. 2015). Experimentally adding the capacity to ferment a new sugar (galactose and raffinose) in *Kluyveromyces lactis* suggests that this shift is relatively easy to attain (Goffrini et al. 2002). Our phenotypic mapping and PS support this idea, suggesting that fermentative versatility is conserved across the phylogeny, but interspersed by frequent shifts.

Data supporting a link between cell size and physiological versatility in yeasts are scarce, as most results relate gene with traits in one or a few species at a time. The best example of cell‐size correlated evolution in unicellular organisms comes from diatoms, where lineages with large cells evolved in salt‐waters, and lineages with small cells evolved in freshwaters, suggesting different evolutionary optima (Nakov et al. 2014). The benefits of small cells are related with high rates of nutrient acquisition and high metabolic intensity (Finkel et al. 2010; Nakov et al. 2014; Wright et al. 2014), whereas large cells could escape predation and avoid other stressors (Raven et al. 2005). The needed physiological capacity to process a larger repertoire of compounds could be linked to cell‐size because: (1) larger cells can also have higher compartimentalization, and (2) larger cells can also have a larger nucleus, which in turn could have an more unfolded genome for transcription (Raven et al. 2005; Connolly et al. 2008; Finkel et al. 2010). However, in yeasts this is not clear and can be only inferred indirectly. For instance, selection pressures for increasing size induce the experimental evolution of multicellular (compartimentalized) flocks (Ratcliff et al. 2015). Also, cells (of *Saccharomyces cerevisiae*) grow larger in glucose than in ethanol (Vanoni et al. 2005). This evidence is intriguing and warrants further confirmatory analyses for the correlated evolution of cell‐size and fermentation capacity (either its diversity or magnitude). Common garden experiments and direct measurements of cell volume would be critical in this aim.

Phylogenetic comparative studies make a number of assumptions, especially when using traits from literature (the most common practice; see reviews in Revell 2009; Rezende and Diniz 2012; Rojas et al. 2013). First, traits are measured without error and under common‐garden conditions. This is obviously a constraint, as phenotypic values are variable (especially in microorganisms), and depend strongly on environmental conditions. However, whereas a common‐garden experiment performed in multicellular animals or plants are very difficult to attain, this is a real possibility in unicellular organisms, which can have five or six generations per day. It is surprising that these combinations of experiments and phylogenetic analyses are not common. A second important assumption, particularly for diversification analysis, is that taxon sampling is complete or at‐least complete, and a random sample of the known diversity. This limitation is inherent to the phylogeny being used, which for the our case seems to be the most complete (studies based on Kurtzman and Robnett phylogeny: Hall et al. 2005; Hagman et al. 2013, 2014; Dashko et al. 2014; Hagman and Piskur 2015; Williams et al. 2015). Although we don't have reasons to think that this phylogeny is biased or unrepresentative, the fact that there exist more than thousands described yeasts species (see Kurtzman et al. 2011) warrants further confirmatory studies, with new, larger phylogenies. Another limitation, especially relevant for adaptive radiation theory, is the possibility of not having chosen an ecologically relevant trait (especially in disparity analysis). We cannot surpass this limitation at this point. Further confirmation of our findings are needed, especially with disparity and PS analysis of traits with direct relevance for fermentation, such as alcohol and CO_2_ production.

In summary, in this article we provide a comparative phylogenetic analysis in yeasts using a number of statistical tools and provocative tests that we hope will inspire other researchers in the field. Whereas our results do not support the idea of adaptive radiation in yeasts, they suggest several patterns that can be further explored (e.g., cell‐size as an evolutionary constraint for metabolic capacity).

## Conflict of Interest

None declared.

## Data Accessibility

Data for this article will be deposited in the Dryad Digital Repository: http://doi:10.5061/dryad.2hf06

## Supporting information


**Data S1.** kurtzToR.csv file.
